# Longitudinal Testing of *Leptospira* Antibodies in Horses Located near a Leptospirosis Outbreak in Alpacas

**DOI:** 10.3390/vetsci9080426

**Published:** 2022-08-12

**Authors:** Charlotte Bolwell, Erica Gee, Brooke Adams, Julie Collins-Emerson, Katherine Scarfe, Shahista Nisa, Emma Gordon, Chris Rogers, Jackie Benschop

**Affiliations:** 1 School of Veterinary Science, Massey University, Palmerston North 4442, New Zealand; 2 IDEXX Laboratories (NZ) ULC, School of Veterinary Science Complex, Massey University, Palmerston North 4442, New Zealand; 3 School of Agriculture and Environment, Massey University, Palmerston North 4442, New Zealand

**Keywords:** horse, *Leptospira* serovars, leptospirosis, New Zealand

## Abstract

**Simple Summary:**

The objective of this study was to look at antibodies in repeated blood samples from horses kept near, or on, a farm where Leptospirosis was diagnosed in a herd of alpacas, resulting in kidney disease and abortion in the alpacas. Blood samples from horses in New Zealand have previously shown approximately 25% have antibodies to Leptospira, although there are few reports of clinical disease. Seven of twelve horses had positive antibody results during the current study, and two horses had high concentrations of antibodies in their blood together with evidence of leptospires in their urine. These results suggest the two horses could have been actively infected with Leptospira, and potentially be at risk of transmitting the disease to humans and other animals on the property. It was not able to be determined if there was a direct association between the positive horses in this study and the outbreak in alpacas. Potentially, there could have been a common exposure for both horses and alpacas, or one group may have infected the other. The potential risk of horses shedding leptospires that could infect humans, or other species, should not be overlooked in New Zealand.

**Abstract:**

The objectives of this study were to determine if horses located near an outbreak of leptospirosis in alpacas had *Leptospira* titres indicative of a previous or current infection and, if so, to determine the magnitude in change of titres over time. Further, the objective was to determine if horses with high titre results were shedding *Leptospira* in their urine. Blood samples were collected from twelve horses located on or next to the farm with the outbreak in alpacas, on day zero and at four subsequent time points (two, four, six and nine weeks). The microscopic agglutination test was used to test sera for five serovars endemic in New Zealand: Ballum, Copenhageni, Hardjo, Pomona and Tarassovi. A reciprocal MAT titre cut-off of ≥1:100 was used to determine positive horses. Seven out of twelve horses (58%) were positive to at least one serovar during one of the time points. Two horses recorded titres of ≥1600, one for both Pomona and Copenhageni and the other for Hardjo, and these two horses were both PCR positive for *Leptospira* in their urine samples. For five out of seven horses, the titres either remained the same or changed by one dilution across the sampling time points. The study confirmed endemic exposure to five endemic *Leptospira* serovars in New Zealand in a group of horses located near a confirmed leptospirosis outbreak in alpacas.

## 1. Introduction

Leptospirosis is a zoonotic disease of worldwide importance. In horses, leptospirosis can result in abortion in pregnant mares, renal disease and the subsequent development of equine recurrent uveitis (ERU) [1,2,3]. History of exposure to *Leptospira* and evidence of shedding the bacteria in urine have been commonly reported in apparently healthy horses [1,2,4]. Worldwide, the seroprevalence of *Leptospira* exposure in horses ranges from 1% to 95%, depending on the country, serovars studied and titre cut-points [2,5,6,7]. In New Zealand, 25% (124/499) of apparently healthy racing and breeding horses sampled were positive to at least one of the *Leptospira* serovars tested [8]. However, there are relatively few clinical reports of leptospirosis in horses in New Zealand.

Due to the temperate climate in New Zealand, horses are commonly kept at pasture year-round [9,10] and co- or alternately grazed with other livestock [11]. Previous studies have identified co-grazing or alternately grazing horses with livestock [8,12], sharing water sources with other livestock and contact with livestock on neighbouring properties (over fences) [13] to be risk factors for *Leptospira* exposure in horses. A recent study reported that livestock on most beef cattle, sheep or deer farms in New Zealand had been exposed to at least one of the serovars routinely tested for in New Zealand [14]. While dairy cattle in New Zealand are routinely vaccinated for *Leptospira* serovars Hardjo and Pomona [15], and commercial pig producers routinely vaccinate for serovars Pomona and Tarassovi [16], vaccination against *Leptospira* in beef cattle, sheep and deer is not common [17]. Unlike in the USA [3], there is no licensed vaccine available for horses in New Zealand.

In October 2017, an outbreak of abortion and renal disease due to *Leptospira* serovar Pomona was confirmed for the first time in a herd of alpacas in New Zealand. The herd consisted of seventy alpacas, including fifty breeding females and twenty crias. All females were tested for *Leptospira* serovars Pomona, Copenhageni and Hardjo. Eleven of the fourteen pregnant females aborted, of which two had paired rising titres for *Leptospira* Pomona, and four aborted fetuses were PCR positive for *Leptospira* DNA. Two crias became systemically ill and developed acute renal disease; one cria subsequently died. The cria that recovered, and an additional twenty-three females, had *Leptospira* Pomona titres of ≥1:3200 [18].

At the time of the leptospirosis outbreak in the alpacas, horses were kept at pasture on, and next to, the case farm. The farms were in the Manawatu region near Massey University, which presented an opportunity to sample the horses located near the outbreak to determine their seropositivity to *Leptospira* over time. Given that co- or cross-grazing horses with other animals were previously identified as risk factors for *Leptospira* seropositivity in horses in New Zealand [8], the objectives of this study were to determine if any of the horses had *Leptospira* titres indicative of a previous or current infection and, if so, to determine the magnitude in change of titres over time. Further, the objective was to determine if horses with high titre results were shedding *Leptospira* in their urine.

## 2. Materials and Methods

The study population of horses were from the same farm as the leptospirosis outbreak in alpacas (Farm 1, *n* = 3 horses), or the farm next to it (Farm 2, *n* = 20 horses). The owners of the horses were contacted to seek their permission to sample the horses and written consent forms were signed by each consenting owner. The horse owners were asked to complete a questionnaire about the management of their horses, whilst the owners of the properties where the horses were kept completed a questionnaire relating to the management of the farms. Demographic information, history of ERU and testing for leptospirosis was collected for the horses, and the farm questions related to possible environmental, other animal or management exposures to *Leptospira* and have been previously described in detail [8]. The horse and property questionnaires were evaluated by peer review and judged to be low risk; approval by the Massey University Human Ethics Committee was not required.

The blood sampling of horses as part of this study was approved by the Massey University Animal Ethics Committee, Massey University, Palmerston North (Protocol number 17/89). The outbreak of abortions in alpacas occurred during August 2017, with leptospirosis confirmed in late October 2017. The sampling of horses took place between November 2017 and January 2018. Blood samples were taken from all horses enrolled in the study at day zero (T1) (Day 15 from confirmation of high *Leptospira* titres in the alpacas) and at four subsequent sampling times (T2–T5) two, four, six and nine weeks from the first sampling date. Blood samples (2 × 10 mL red top vacutainer and 20 g vacutainer needle; (Becton Dickenson Limited (BD), Auckland, New Zealand) were collected once from the jugular vein of each horse into 10-mL vacuum tubes. Samples were placed in a cooled and insulated transport container or refrigerated from 4–8 °C until submitted to a commercial veterinary diagnostic laboratory (IDEXX Laboratories Ltd., Massey University, Palmerston North) for serum extraction and testing. Most samples were submitted within three days of sampling, except for nine samples taken in T5 that were centrifuged at 1300× *g* for 10 min and sera frozen and stored at −80 °C for one week.

A microscopic agglutination test (MAT) as described by Faine [19] was used to detect antibodies against *Leptospira interrogans* serovars Pomona and Copenhageni and *Leptospira borgpetersenii* serovars Ballum, Hardjo and Tarassovi; no other serovars were routinely tested for as they are considered exotic to New Zealand [20]. Another serovar, Balcanica, that is genetically very similar to Hardjo, is also known to be endemic in New Zealand. It is indistinguishable from Hardjo in MAT; however, the serovar is maintained within the possum population and is incidental and not transmissible within other host populations [21]. For this reason, positive Hardjo MAT titres in the horses were assumed to be Hardjo.

As described previously [8], serum samples were diluted 1/6.25 in phosphate-buffered saline (PBS; Lorne-buffered saline tablets 0.9% NaCl, Lorne Laboratories Ltd., Reading, UK), and doubling dilutions were made to obtain a final series ranging from 1:25 to 1:12,800, inclusive of the addition of the live antigen. Antigen strains were purchased from the Institute of Environmental Science and Research (ESR), Porirua, New Zealand. Standard antisera were from the World Organisation for Animal Health (OIE), Reference Laboratory for Leptospirosis (Amsterdam, The Netherlands). The end point titre was recorded at the highest dilution where at least 50% agglutination occurred, and all tests were carried out by the same technician (KS).

A reciprocal MAT titre cut-off of ≥100 was used to determine positive horses [22]. Results are summarised as the number and percentage of horses that were negative, had serum MAT titres of 25 or 50, and ≥100 for all serovars tested across the five time points. The titre results across each serovar and time point are reported individually for horses determined to be positive at any time point to any serovar.

Horses that had high titres of >400 [23] were given a full clinical exam at the Massey University Equine Veterinary Clinic. As part of the clinical exam, a urine sample was collected via sterile urinary catheterization (mares: PVC tubing, 8.6 mm diameter, 60 cm length; geldings: 28 Fr silicone tubing, 150 cm length, Surgivet^®^ EUC28150). Using a 60-mL catheter tip sterile syringe, 60 mL of urine was extracted and decanted into sterile pottles and submitted to the Molecular Epidemiology and Public Health Laboratory (mEpiLab, Hopkirk Institute, School of Veterinary Science, Massey University, Palmerston North, New Zealand) within one hour of collection. Shedding status of the horse (presence of leptospires in the urine) was determined using polymerase chain reaction (PCR). All gDNA from urine samples were extracted using the QIAamp^®^ DNA mini kit (Qiagen; Hilden, Germany) according to the manufacturer’s instructions and subjected to PCR with primers glmU_DW_F (5′-CCGTATGAAAACGGATCAGCC-3′) and glmU_DW_R (5′-ATTCTCCCTGAGCGTTTTGATTTC-3) using the conventional PCR protocol previously described [24]. All PCRs included positive controls consisting of purified *Leptospira* DNA from *L. borgpetersenii* serovar Hardjo type bovis and *L. interrogans* serovar Pomona. Invitrogen UltraPure™ Distilled Water was used as negative controls. The amplicons were analysed by gel electrophoresis in a 1% agarose gel stained with RedSafe to visualise the 523 bp product.

## 3. Results

### 3.1. Horse and Farm Information

**Figure 1:**
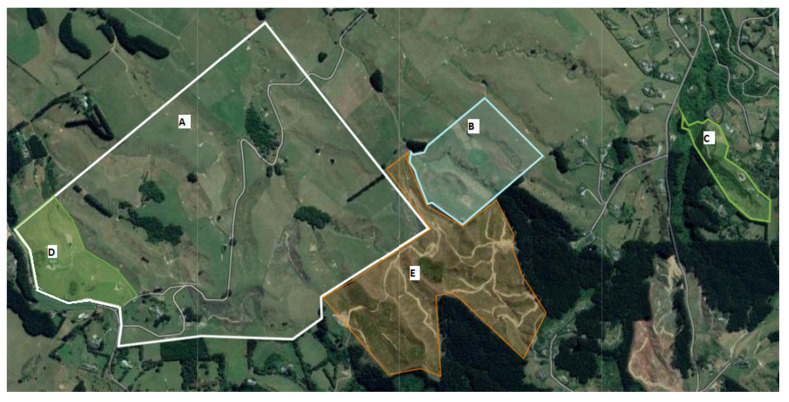
Satellite map showing the location of the two farms where a group of horses were blood sampled as part of testing for leptospiral antibodies after a leptospirosis outbreak in alpacas. Location of Farm 1 containing alpaca and horses (**C**), Farm 2 (**A**) and grazing location of horses on Farm 2 (**D**), land where alpaca were located part of the year (**B**) and access to riding trails for horses (**E**).

Overall, consent was gained to include twelve horses in the study population from Farm 1 (*n* = 3) and Farm 2 (*n* = 9) (Table 1). Nine horses were sampled at all five time points; the T5 sample was missing for three of the horses as the owner was absent. The demographics of the horses sampled are shown in Table 1. None of the horses had a history of being tested for *Leptospira* or a history of ERU. The horses were located on two farms adjacent to each other, with Farm 1 being the location of the alpaca leptospirosis outbreak (Figure 1). At the time of sampling, the horses on Farm 1 were not paddocked together but had been during the previous 12 months. Nine horses, across five owners, were located on Farm 2. On Farm 2, each owner generally had a paddock each for their horses to share, however, horses 546 and 547 had shared a paddock in the last 12 months. Horses on Farm 2 were frequently ridden together, and the owners had access to the farm and neighbouring forest for riding.

Results of the farm survey are shown in Table 2. Neither property had a natural water source or history of flooding; stock drinking water was either rain water collected into tanks (Farm 1) or bore water (Farm 2), pumped to troughs. Farm 2 reported no signs of wildlife on the property apart from rabbits. Both farms had cats, dogs and sheep on the property, with Farm 2 also having beef cattle. No animals on Farm 2 were reported as being vaccinated for leptospirosis, whilst the dogs on Farm 1 were vaccinated. On Farm 1, horses were grazed alternately, had contact over the fence and shared the same water source with sheep and alpaca. Horses on Farm 2 shared the same water source with cattle and were grazed alternately, had contact over the fence and shared the same water source as sheep (Table 2).

### 3.2. Serology

The number and percentage of horses by titre level, time point and serovar is shown in Table 3. Positive results were recorded for Pomona and Copenhageni by two or three horses, two or one horse for Hardjo and one horse for Ballum and Tarassovi at each of the time points (Table 3). Peak titres were recorded for Pomona and Hardjo (≥6400). The highest titre for serovar Copenhageni was 1600 recorded at T1, whilst the highest titre for Ballum and Tarassovi was 200 and 100, respectively.

Seven out of twelve horses (58%) were positive to at least one serovar during one or more of the time points. Horse 543 had a Pomona titre of <100 at T1 and became positive at T2, returning two further positive samples in T3 and T5 (Table 4). Two horses recorded titres of ≥1600 at T1; horse 540 for Pomona and Copenhageni and horse 547 for Hardjo; these horses also recorded the peak titres for these serovars (Table 4). Two horses (538 and 540) were positive to more than one serovar, with horse 538 returning positive results for Pomona, Copenhageni and Tarassovi. For five out of seven horses, the titres either remained the same or changed by one dilution across the sampling time points.

### 3.3. Clinical Exam and PCR

Due to high titre results at T1, clinical exams, including an ophthalmic exam and urine collection, were performed on two horses from Farm 2 (540 and 547, Table 4) at T2. Both horses were PCR positive on urine samples but found to be clinically normal upon examination.

## 4. Discussion

This study investigated longitudinal changes in *Leptospira* titres in healthy horses. The study was initiated due to the horses’ location to an outbreak of leptospirosis in alpacas. The results showed widespread exposure to five serovars tested in this group of horses. Over half of the horses were positive to at least one serovar tested (based on a titre of ≥100), which was higher than the 25% previously reported in a serological survey of apparently healthy racing and breeding horses in New Zealand [8]. Two horses were positive to more than one serovar, which agrees with previous findings reporting 19% of positive horses were positive to more than one of the five serovars tested [8].

In this study the highest titres were recorded for serovar Pomona, which was also detected as part of the outbreak in the alpacas [18]. Although data on prevalent serovars in horses in New Zealand is limited, Pomona has been linked to cases of sporadic abortions [25,26], a case of renal disease and two cases of ERU [27,28]. High titres were also observed in this study for Hardjo, and for Copenhageni in the horse that also showed high titres to Pomona. It is likely that the infecting serovar for this horse was Pomona, and the Copenhageni titres may be due to a cross-reaction. Infections in horses due to Pomona have been noted to produce antibodies that cause cross-reactions with other serovars [29], producing titres that are usually lower than the infecting serovar and decline quickly [22,29,30]. All three horses with Pomona positive titres also had titres to other serovars at lower levels. The infecting serovar cannot be definitively determined for all horses in the current study and further work is required to determine if one serovar may be more prevalent in (associated with) horses in New Zealand.

An advantage of this novel study was following the change in *Leptospira* titres longitudinally for seropositive horses. Fluctuations in individual titres occurred over the five sampling points; one horse seroconverted and titres for five horses changed by either one dilution or stayed the same between subsequent time points. These results suggest that whilst a one-off test may indicate history of exposure, a one-off “snapshot” test has limited value in diagnosing current infection, which is consistent with previous reports [2]. Further, variations in MAT titres by one dilution could be due to differences associated with test conditions, such as concentration and age of the antigen and a degree of subjectivity around test reading. Therefore, a four-fold rise in titres for paired samples is needed to determine an active or current infection [2,22], which was not detected in this study.

Given the design and sampling time frame of this study, it is not possible to accurately determine if there was an association between the alpaca outbreak and the positive horses sampled in this study, or the direction of any association or if there was common exposure from another source, such as wildlife. Potentially, there could have been a common exposure for both horses and alpacas, and outbreak may have firstly occurred either in horses or in alpacas, leading to infection in the other species. Due to the timing of the samples in the horses, the serostatus of the horses before the outbreak is not known. It is possible that there was a common exposure that occurred prior to the outbreak, or at the same time as the alpacas were exposed, as *Leptospira* titres may be detected in horses for several years after infection [22,29]. However, the horses were sampled approximately twelve weeks after the first abortion in the alpacas occurred, and *Leptospira* antibodies in horses have been shown to develop as early as five days after infection [29]. Additionally, two horses with the highest titres were urinary PCR positive, indicating they were shedding leptospires in their urine. Culture was not performed, so it could not be confirmed these were active infections, despite these horses being clinically normal on evaluation.

The management of horses at pasture has been previously linked to an increased risk of exposure to *Leptospira* [8,31]. Although risk factors were not directly analysed in the current study, several management factors related to livestock on the two properties investigated have previously been identified as increasing the risk of exposure to horses [8,13]. Horses on both properties shared a water source, had contact over the fence and were grazed alternately with sheep. Horses on Farm 2 shared a water source with cattle, and horses on Farm 1 shared a water source, had contact over the fence and were grazed alternately with alpaca. However, specific risks associated with contact with alpacas on a property with or next to horses have not been previously investigated. The outbreak of leptospirosis in alpacas was the first reported in New Zealand [18], suggesting future studies may need to consider the possible impact of contact of alpacas with horses, other livestock or people and the role that alpacas may play in the epidemiology of leptospirosis in New Zealand.

Leptospirosis is a significant concern for human health in New Zealand and one of the main risk factors is occupational exposure, particularly farmers and meatworkers [32]. Two horses in the current study were shedding leptospires, which may have posed a zoonotic risk to those working with the horses. However, there are no specific studies investigating the risk of exposure to people who own or work with horses in New Zealand. Two respondents in a previous seroprevalence survey of horses in New Zealand reported a diagnosis of leptospirosis in family members [8], but the study did not specifically investigate exposure in the horse owners. Interim findings from a current New Zealand case control study [33] reported a crude odds ratio of 1.7 [95% CI: 0.7–4.1] for contact with horses in the four weeks before illness onset (cases) or interview (controls). It is noteworthy that 12/67 cases and 17/150 controls reported contact with horses [34]. The prevalence of exposure and reported cases of leptospirosis in horse workers and owners in New Zealand requires further investigation.

The study confirmed exposure to five endemic *Leptospira* serovars in New Zealand in a group of horses located near a confirmed leptospirosis outbreak in alpacas. Despite being clinically normal, two horses were found to be shedding leptospires in their urine, suggesting current infection. These horses may pose a zoonotic risk to people or other animals on the properties. The management of the horses at pasture was consistent with previously identified risk factors for *Leptospira* exposure, such as contact with other livestock

## Figures and Tables

**Table 1:** Details of the horses tested for leptospiral antibodies, located near an outbreak of leptospirosis in alpacas. Information on the horses was collected at the time of testing.

Horse	Owner	Farm	Age ^1^ (Years)	Breed	Sex	Time Resident on Property	History of Leptospirosis ^2^	History of Uveitis
536	1	1	8	Warmblood	Mare	8 years	No	No
537	1	1	6	Warmblood	Mare	1.5 years	No	No
538	1	1	27	Warmblood	Mare	10 years	No	No
539	2	2	14	Thoroughbred	Mare	1 year	No	No
540	2	2	7	Warmblood	Mare	1 year	No	No
541	2	2	13	Warmblood	Mare	1 year	No	No
542	3	2	14	Warmblood	Gelding	15 months	No	No
543	3	2	15	Warmblood	Mare	1.5 years	No	No
544	4	2	7	Warmblood	Mare	3 years	No	No
545	4	2	17	Warmblood	Mare	3 years	No	No
546	5	2	14	Thoroughbred	Mare	10 months	No	No
547	6	2	14	Thoroughbred	Gelding	1 year	No	No

^1^ Age at sampling; ^2^ History or testing for *Leptospira* exposure or infection.

**Table 2:** Results of the farm questionnaire conducted with the owners of the farms where horses were located during sampling for leptospiral antibodies, which were located near to outbreak of leptospirosis in alpacas.

Variable	Farm 1	Farm 2
Natural water source on property	No	No
Flooding on property in the last 12 months	No	No
Evidence of wildlife on the property		
Rats	Yes	No
Mice	Yes	No
Possums	Yes	No
Hedgehogs	Yes	No
Rabbits	Yes	Yes
Mustelids	Yes	No
Animals on property		
Cats	Yes	Yes
Dogs	Yes	Yes
Pigs	No	No
Goats	No	No
Dairy cattle	No	No
Beef cattle	No	Yes
Sheep	Yes	Yes
Deer	No	No
Alpaca	Yes	No
Animals on the property vaccinated for lepto ^1^	Yes—dogs	No
Contact with other animals		
Graze horses same time as cattle	No	No
Graze horses alternately with cattle	No	No
Horses share water source with cattle	No	Yes
Contact with cattle over fence	No	No
Graze horses same time as sheep	No	No
Graze horses alternately with sheep	Yes	Yes
Horses share water source with sheep	Yes	Yes
Contact with sheep over fence	Yes	Yes
Graze horses same time as alpaca	No	No
Graze horses alternately with alpaca	Yes	No
Horses share water source with alpaca	Yes	No
Contact with alpaca over fence	Yes	No

^1^ Were other animals on the property vaccinated against *Leptospira*. Horses are not vaccinated against *Leptospira* in New Zealand.

**Table 3:** Number of horses by MAT titre for each serovar at each sampling time point in a group of horses tested for *Leptospira* antibodies, which were located near to outbreak of leptospirosis in alpacas.

Serovar	Time Point	MAT Titre										Total Horses
		<25	25–50	100	200	400	800	1600	3200	6400	12,800	
Pomona	1	8	2	0	1	0	0	0	0	0	1	12
	2	7	2	1	1	0	0	1	0	0	0	12
	3	9	0	1	1	0	0	0	1	0	0	12
	4	8	2	1	0	0	0	0	1	0	0	12
	5	5	2	1	0	0	0	0	0	1	0	9
Hardjo	1	8	2	0	1	0	0	0	0	1	0	12
	2	9	1	1	0	0	0	1	0	0	0	12
	3	9	1	1	0	0	0	0	0	1	0	12
	4	9	2	0	0	0	1	0	0	0	0	12
	5	7	0	1	0	0	0	1	0	0	0	9
Copenhageni	1	5	5	0	0	1	0	1	0	0	0	12
	2	8	2	0	1	1	0	0	0	0	0	12
	3	9	1	1	1	0	0	0	0	0	0	12
	4	8	1	2	1	0	0	0	0	0	0	12
	5	6	1	0	2	0	0	0	0	0	0	9
Ballum	1	8	3	0	1	0	0	0	0	0	0	12
	2	10	2	0	0	0	0	0	0	0	0	12
	3	10	1	1	0	0	0	0	0	0	0	12
	4	10	2	0	0	0	0	0	0	0	0	12
	5	8	1	0	0	0	0	0	0	0	0	9
Tarassovi	1	7	5	0	0	0	0	0	0	0	0	12
	2	9	3	0	0	0	0	0	0	0	0	12
	3	8	4	0	0	0	0	0	0	0	0	12
	4	11	0	1	0	0	0	0	0	0	0	12
	5	9	0	0	0	0	0	0	0	0	0	12

**Table 4:** MAT titre results by serovar and time point for positive horses, those with at least one MAT titre of ≥100 to any serovar at any time point, in a group of horses tested for *Leptospira* antibodies, which were located near to outbreak of leptospirosis in alpacas.

		Serovar				
Horse	Time Point	Pomona	Hardjo	Copenhageni	Ballum	Tarassovi
538	1	200	50	50	0	50
	2	200	25	25	0	50
	3	200	25	25	0	0
	4	100	25	100	0	100
	5	-	-	-	-	-
540	1	12,800	25	1600	0	0
	2 *	6400	0	400	0	0
	3	3200	0	200	0	0
	4	3200	0	400	0	0
	5	6400	0	200	0	0
543	1	0	0	25	0	0
	2	100	0	0	0	0
	3	100	0	0	0	25
	4	25	0	0	0	0
	5	100	0	0	0	0
544	1	0	200	0	0	25
	2	0	100	0	0	0
	3	0	100	0	0	0
	4	0	50	0	0	0
	5	0	100	0	0	0
545	1	0	0	400	0	25
	2	0	0	200	0	0
	3	0	0	100	0	25
	4	0	0	100	0	0
	5	25	0	200	0	0
546	1	0	0	0	200	0
	2	0	0	0	50	0
	3	0	0	0	100	0
	4	0	0	0	50	0
	5	0	0	0	50	0
547	1	0	6400	0	25	0
	2 *	0	1600	0	0	0
	3	0	6400	0	0	0
	4	0	800	0	0	0
	5	0	1600	0	0	0

T5 sample was not obtained for horse 538; * urine PCR positive.

## Data Availability

The data presented in this study are available upon request from the corresponding author.
